# Experimental Study of Two-Bite Test Parameters for Effective Drug Release from Chewing Gum Using a Novel Bio-Engineered Testbed

**DOI:** 10.3390/biomedicines13081811

**Published:** 2025-07-24

**Authors:** Kazem Alemzadeh, Joseph Alemzadeh

**Affiliations:** 1 (ESDI) Bionics and Bioengineering Research Group, Faculty of Engineering, School of Electrical, Electronic and Mechanical Engineering, University of Bristol, Bristol BS8 1TR, UK; 2 Alumnus of the Cardiff University, School of Engineering, Cardiff University, Cardiff CF24 3AA, UK; jalemzadeh@googlemail.com

**Keywords:** humanoid chewing robot, biomimetic chewing chamber, occluded molars, effective mastication, crush/shear ratio, dental morphology and jaws physiology, FMA and BA angles, two-bite test, medicated chewing gum, mechanochemistry

## Abstract

**Background**: A critical review of the literature demonstrates that masticatory apparatus with an artificial oral environment is of interest in the fields including (i) dental science; (ii) food science; (iii) the pharmaceutical industries for drug release. However, apparatus that closely mimics human chewing and oral conditions has yet to be realised. This study investigates the vital role of dental morphology and form–function connections using two-bite test parameters for effective drug release from medicated chewing gum (MCG) and compares them to human chewing efficiency with the aid of a humanoid chewing robot and a bionics product lifecycle management (PLM) framework with built-in reverse biomimetics—both developed by the first author. **Methods**: A novel, bio-engineered two-bite testbed is created for two testing machines with compression and torsion capabilities to conduct two-bite tests for evaluating the mechanical properties of MCGs. **Results**: Experimental studies are conducted to investigate the relationship between biting force and crushing/shearing and understand chewing efficiency and effective mastication. This is with respect to mechanochemistry and power stroke for disrupting mechanical bonds releasing the active pharmaceutical ingredients (APIs) of MCGs. The manuscript discusses the effect and the critical role that jaw physiology, dental morphology, the Bennett angle of mandible (BA) and the Frankfort-mandibular plane angle (FMA) on two-bite test parameters when FMA = 0, 25 or 29.1 and BA = 0 or 8. **Conclusions:** The impact on other scientific fields is also explored.

## 1. Introduction

Over the last four decades, masticatory apparatus has rapidly developed. A review of the literature demonstrates that apparatus with an artificial oral environment is of interest to fields including (i) dental science, where the focus is on material testing and failure points [1,2,3]; (ii) food science that focuses on bolus breakdown for flavour release [4,5,6,7], (iii) the pharmaceutical industry for drug release [8,9]. However, instruments that closely mimic human chewing and oral conditions have yet to be realised [10].

Regardless of the applications, during the design process of bio-inspired products there is a trade-off [11] when seeking alternative designs for optimum solutions, especially in complex systems. This means that improving one aspect of a design might necessitate compromising on another. Trade-offs are inherent features of many biomechanical systems and are often seen as evolutionary constraints. The structural decoupling and abstraction of the relationship may provide a way to overcome those limits in some systems but not for the form–function structures that transmit large forces, such as mammalian mandibles [12]. A brief, yet critical, review of these trade-offs is listed below for in vitro apparatus within dental/food science and the pharmaceutical industry.

Within dental science regarding wear and microwear, a study by Heintze et al., 2019 [13] concluded that (i) mimicking the entire masticatory cycle with all possible movements of the lower jaw is not necessarily the best approach, (ii) a machine that applies a force in only one direction is also inadequate. Krueger et al., 2021 [14] highlighted the uncertainties surrounding the mechanisms underlying its formation due to a lack of standardisation across in vitro studies. Daegling et al., 2016 [15] mentioned that utilising various chewing simulators or mechanical testing systems may help. However, these studies are utilising different chewing simulators, methodologies for tooth preparation and species used in analysis which inhibits effective comparisons across studies.

Within food science on aroma release, the recent review by Pu et al., 2024 [16] summarised the latest developments in aroma release from food to the retronasal cavity, aroma release and delivery influencing factors and aroma perception mechanisms. They identified that it is necessary to develop more accurate in vitro equipment for simulating the procedures during oral processing. Their study looked at four chewing simulators developed by [6,10,17,18] for food oral processing and they suggested that intelligent chewing simulators are key to establishing a standard analytical method.

Regarding the physiological structures of masticatory simulators and the oral cavity, Guo et al., 2024 [19] produced a comprehensive review of systematic designs from the past 15 years that highlighted the current limitations of these simulators and stated that there is no established standard method and criteria for evaluating their biomimetic components. Their work suggested that it is essential to introduce innovative equipment and testing methods for standardising chewing efficiency, plus criteria for evaluating the biomimetic components of those simulators.

Regarding the effects of dental morphology, form–function and comminution, Walker et al., 2022 [20] investigated the effect of these factors with attention to comminution chewing experiments in catarrhines. Molar intercuspation was used as a tool to perform different food processing actions such as crushing and shearing. Their study reviewed the state of the art chewing experiments and investigated the effects of molar morphology on chewing efficiency with one pair of opposite upper and lower second molars. The chewing simulator used in their study was developed by Salles et al., 2007 [6] for food fracturing which is equipped with two dental replica rings having equal size teeth as human jaws. The simplification of humans’ jaws (i.e., as a trade-off) was apparent, although tooth size has an influence on the fragmentation of certain types of food, in terms of mechanics [21,22,23]. They concluded that due to the limitations of the chewing simulator, the design of a new chewing simulator is underway that will improve the emulation of natural masticatory processes. Other simulators under development [10,14,24,25] may allow further studies on the form–function relationships of teeth.

Within the pharmaceutical industry, drug release from a medicated chewing gum (MCG) is an advanced drug delivery method with a promising future [26,27]. Chewing gum is one of the modern approaches to oral transmucosal drug delivery and is a useful means for systemic drug delivery. The advantages of MCG over other oral mucosal drug delivery systems are the possibility of controlled drug release over an extended time and the potential to improve the variability in drug release and retention times. There are other advantages to delivering pharmaceutical drugs through chewing gum rather than conventional tablets that are usually swallowed with water. A major advantage of delivering pharmaceutical drugs through chewing gum, rather than conventional tablets, lies in the absorption of the drug through the oral mucosa. This avoids metabolism in the gastrointestinal tract and reduces first-pass metabolism. A lower concentration of the drug can be prescribed because it has a greater bioavailability and a faster onset of action when it is absorbed into the blood circulation in this way [28,29]. In addition to the health benefits, the potential reduction in the active pharmaceutical ingredient (API) can both reduce costs and increase the availability and accessibility of MCGs across the globe. For example, MCGs containing casein phosphopeptide-amorphous calcium phosphate (CPP-ACP) were shown to protect against tooth decay [30,31] which was reported to affect 2.4 billion people worldwide in 2010 [32] and more than 35% globally in 2020 [33]. Other MCG examples contain aspirin, caffeine, dimenhydrinate, vitamin C and chlorhexidine [28]. The global markets for aspirin and vitamin C are worth USD 3bn and USD 1.8bn, respectively [34,35], meaning that a reduction in API of 10–20% could result in savings of USD 0.5bn per annum. Recent studies suggest that curcumin (CUR) chewing gums have potential therapeutic benefits for head and neck cancer patients [28,36,37], and angiotensin-converting enzyme-2 chewing gums have been shown to decrease oral virus transmission and the infection of SARS-CoV-2 [38].

The potential and the health benefits of MCGs [39] have not yet been fully exploited [40] due to the following:

(1) The inherent technical and scientific challenges in realising in vitro apparatus to test drug release prior to human studies. These challenges arise from the need to replicate the intricate jaw physiology and human oral environment with masticatory motions and forces, and the flow of saliva over the artificial gum surface necessary for the release of the API from the MCG [10]. The Frankfort-mandibular plane angle (FMA) and Bennett angle (BA) are important cephalometric measurements that reflect the interplay between jaw structure, dental morphology and overall facial form–function. The FMA indicates the vertical jaw relationship and the BA is related to mandibular movement during lateral excursions, which reflects the temporomandibular joint’s (TMJ) influence on jaw function. The FMA and BA are crucial in understanding chewing because they influence jaw movement and the way teeth come together during mastication. The FMA impacts on bite force and tooth arrangement. The BA is vital for proper occlusal relationships and the design of prosthetic restorations. The FMA and the BA are critical parameters in understanding the biomechanics of chewing (i.e., crushing, shearing and grinding), impacting both the natural occlusion and the design of prosthetic restorations.
(2) The complexity of their formulation, lack of acceptable testing methods and intricacy of their manufacturing. This is due to the scarcity of studies concerning the evaluation of the mechanical properties of MCGs [28,37,41,42].

The current state of the art described in the European Pharmacopeia [43] includes apparatus A [8] and B [9] which were developed by Christrup and Møller 1986 [8] and Kvist et al., 1999, respectively [9], for in vitro drug release testing [28]. However, they yield inconsistent results and fail to accurately simulate complex chewing motions and forces. Moreover, they cannot combine mastication, saliva and chewing gum together to mimic human oral conditions to allow for the measurement of the release of APIs effectively from MCGs and thus are not widely accepted or FDA-approved for this reason [10,44,45,46,47].

A review of methods A and B by Stomberg et al., 2018 [44] indicated that they simplify mastication to a stamping procedure between two flat-faced, optionally rotating pistons. They developed a 45° gliding steel unit with a four-cylindrical-cusped molar [44], which attempted to imitate the occlusal plane of natural dentition with the simulator squeezing the gum with a pre-set deformation force [44]. However, despite the authors’ valiant attempts, the four-cylindrical-cusped molar tooth apparatus had limited anatomical accuracy, and the occlusal surface geometries and mastication were very simplified [44].

A more recent review of the A and B apparatus by Externbrink et al., 2019 [46] highlighted a need for new testing methodologies to evaluate the performance of potential abuse-deterrent opioid products by chewing. They mentioned that apparatus B has been described in the scientific literature and is commercially available (Erweka release tester, Erweka GmbH, Langen, Germany). They used apparatus B in their study, which indicated that the chewing methodology evaluated in their work may provide a useful in vitro tool to characterise chewing resistance and API release properties. However, apparatus B provides a simplified and standardised model of the human jaw with limited capability to fully mimic the complex physiological conditions in the oral cavity during chewing.

Alemzadeh et al., 2020 created a novel chewing robot with built-in humanoid jaws as an artificial oral environment with temperature control and artificial saliva, which was capable of closely replicating human chewing motion [10]. This allowed for the measurement of xylitol release from commercially available chewing gum which was quantified following both in vitro and in vivo mastication. The chewing robot demonstrated a similar release rate of xylitol as human participants [10].

Currently, there is no information that exists on the morphology of occluded pairs of maxillary and mandibular molars in any of the bio-inspired chewing simulators. Specifically, no biomimetic standard design processes and their associated tools could be found that stated how accurately they were abstracted from a biological pair and biomimetically modelled and realised, as highlighted by Guo et al., 2024 in their comprehensive review in 2024 [19].

In this respect, Alemzadeh [48] proposed a bionics product life cycle management (PLM) methodology with built-in reverse biomimetics for innovative product development. The novel PLM framework utilising bionics aimed to aid knowledge transfer from biology to engineering to maintain the accuracy and integrity of form–function relationships during the processes of identification and knowledge transfer between anatomical landmarks and biological structures. The proposed framework supported by technical biology, as an enabling method for abstracting the geometrical biological structures to obtain design intents, removes the limitations of the biomimetic processes facing engineering. The study systematically illustrated the knowledge transfer methodology in a step-by-step manner to analyse and reveal the constructional design and working principles of large-scale, human skeletal biological systems in nature. Alemzadeh’s study [48] presented the comprehensive processes of bionic design and biomimetic modelling, simulation, optimisation and validation techniques necessary for a drug-releasing chewing robot and an anthropometric prosthetic hand.

The investigation herein explores the vital role of jaw physiology, dental morphology and form–function on two-bite tests, also known as texture profile analysis (TPA). While TPA is a standard method for determining textural or mechanical properties in food science [49], it is an unofficial product quality test in the pharmaceutical industry for drug release of solid/semi-solid oral dosage forms, such as tablets, chewable tablets, MCGs and others [28,36,37,41,50,51]. In the latest review in 2023 by Bogdan et al., 2023 [51], texture analysis was identified as a versatile but challenging tool, due to different experimental conditions, the choice of testing protocol and parameters. Their study provided guidelines for the use of a universal testing system and texture analysers with their parameter set-ups for pharmaceutical products such as tablets and chewable tablets.

In this study, a novel, bio-engineered two-bite testbed with compression and torsion capabilities was created for two universal testing machines to investigate TPA parameters for evaluating the mechanical properties of MCGs [28,37,41]. Moreover, it compares the testbed to human chewing efficiency with the aid of a humanoid chewing robot and a bionic-related PLM framework with built-in reverse biomimetics—both developed by Alemzadeh [10,48]. The testbed integrates an occluded pair of maxillary and mandibular molars used in the humanoid chewing chamber [10]. The bionics PLM framework [48] is incorporated to address the current issues surrounding the instrumentation to conduct two-bite tests [52] and inconsistencies that currently exist with in vitro masticatory apparatus, as analysed in Section 1.

Experimental studies were conducted to systematically analyse bio-inspired mastication and to probe the form–function relationships between biting force and crushing/shearing aspects to understand chewing efficiency in relation to mechanochemistry and power stroke for disrupting mechanical bonds. A discussion is given on the effect that different molar morphologies, the Frankfort-mandibular plane angle (FMA) and Bennett angle (BA) have on TPA parameters. Finally, the impact on other scientific fields such as dental science, food science and the pharmaceutical industries for drug release is also explored.

## 2. Materials and Methods: The Novel Two-Bite Bio-Engineered Testbed

### 2.1. Design and Modelling of Maxillary–Mandibular–Bio Testbed

**Figure 1:**
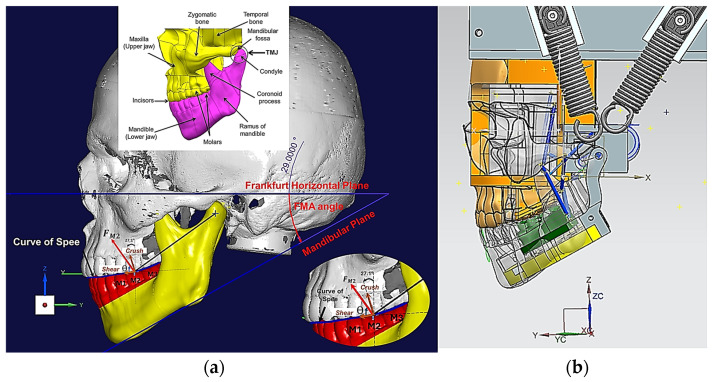
(**a**) 3D CAD skeletal features of the human jaw, the FMA and Curve of Spee with a bite force and crush/shear represented on 2nd molar (M2); (**b**) 3D CAD sagittal view of humanoid chewing robot with removable chewing chamber created from biological pair.

The novel testbed is based on design methodology principles from bionics proposed by Alemzadeh [48], allowing for accurate comminution and molars intercuspation to investigate the biomechanics of chewing sequences, crushing, shearing and grinding. The testbed uses the exact copy of an occluded pair of maxillary and mandibular molars which are built into the artificial oral environment that has been validated with human participants [10] prior to this investigation, as shown in Figure 1. The universal testing machines used were the 50KN 2580 Static Load Cell (Instron, High Wycombe, Buckinghamshire, UK) and Amslet HCT 25 (Zwick-Roell, Ulm, Germany). The latter is capable of performing human chewing frequency (a 49.5 mm/s compression speed), whilst the former is not, so a speed of 10 mm/s was also tested.

The biomimetic design principles of the drug-releasing chewing robot [48] are based on the engineering of dental occlusion principles [53,54,55,56,57] and physiological features [58] associated with the relationships between tooth form and chewing motions [59] that define three types of food processing, shearing, crushing and grinding [23,59,60,61,62], which neither apparatus A nor B fully take into consideration.

For optimum drug release from chewing gum, the crush/shear ratio and power stroke are used for effective chewing [60,61,62] to disrupt mechanical bonds through the biomechanics of chewing sequences [59,60,61,62]. This considers the Frankfort-mandibular plane angle (FMA), Bennett angle (BA) of the mandible [63] and Andrews’s six key principles to normal occlusion [64] and occlusal curvatures, such as the Curve of Spee in the design process [65,66,67,68,69]. These include molar relationship, crown angulation, crown inclination, no rotations, no spaces and flat occlusal planes [64].

**Figure 2:**
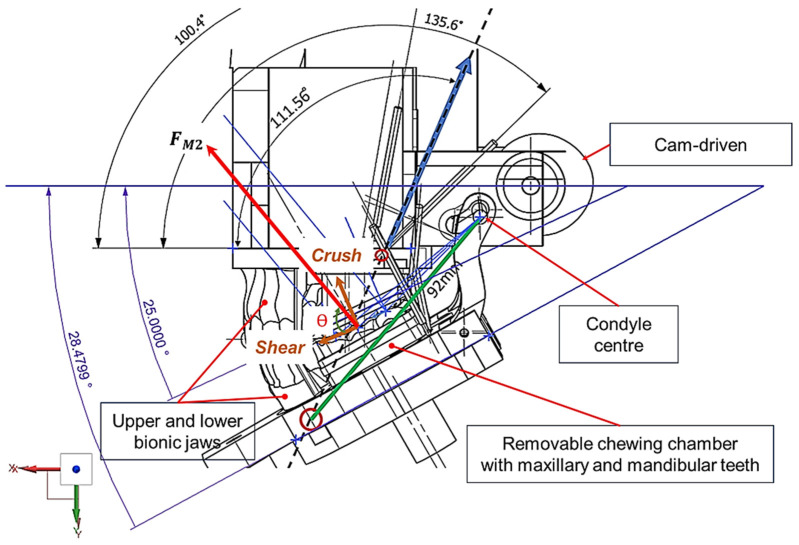
Three-dimensional kinematic diagram of humanoid jaws showing the removable chewing chamber with occluded mandibular and maxillary molars (M1, M2 and M3) and labels on M2.

**Figure 3:**
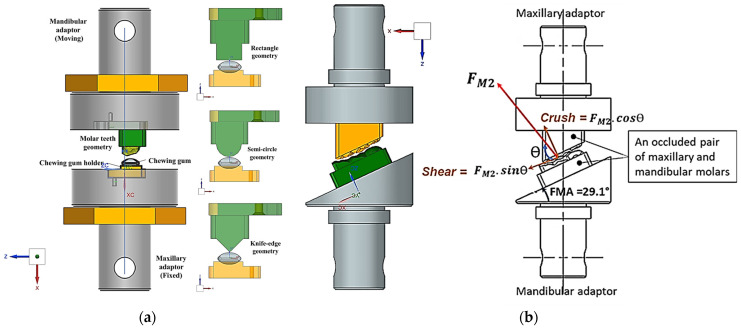
Three-dimensional CAD bio-engineered testbed for Instron compression testing machine. (**a**) Bio-engineered testbed with the 0° FMA for four different tooth designs integrated in the maxillary and mandibular adaptors. (**b**) Bio-engineered testbed with the 29.1° FMA and occluded pair of mandibular and maxillary molars (M1, M2 and M3) and labels on M2.

**Figure 4:**
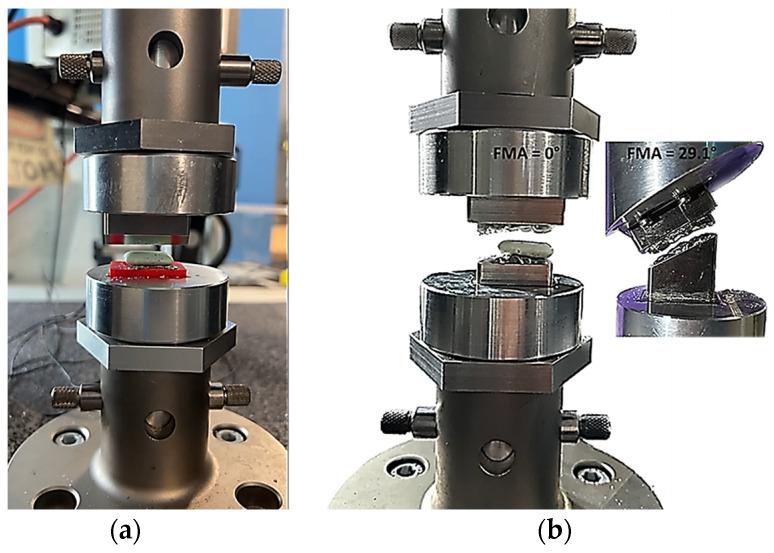
Photographs of the bio-engineered testbed set-up with the 0° and 29.1° FMAs for an Instron compression testing machine. (**a**) Shows the knife-edge geometry design; (**b**) shows occluded pair of mandibular and maxillary molar design.

**Figure 5:**
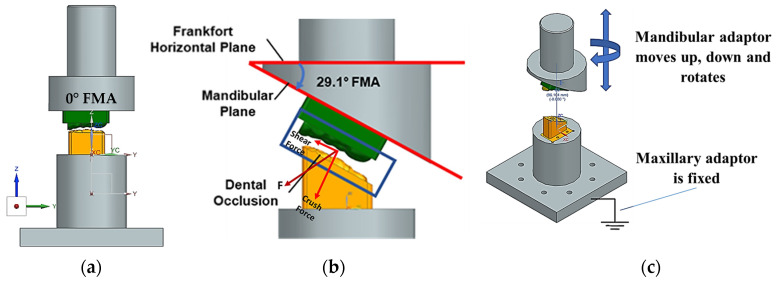
Three-dimensional CAD bio-engineered testbed for Zwick–Roell compression and torsion testing machine. (**a**) and (**b**) show bio-engineered testbed with the 0° and 29.1° FMAs and FHP and MP planes, respectively; (**c**) illustration of the principle of the machine’s motion with the 29.1° FMA, 8° BA and occluded pair of mandibular and maxillary molars.

**Figure 6:**
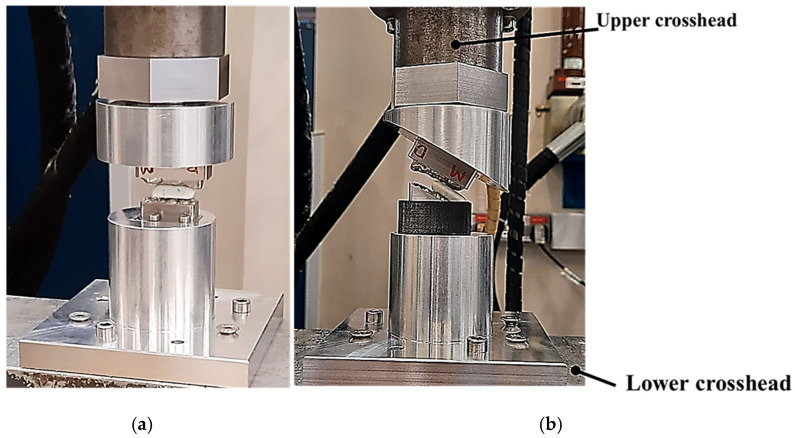
Photographs of the bio-engineered testbed set-up with the 0° and 29.1° FMAs and occluded pair of mandibular and maxillary molars for Zwick–Roell compression and torsion testing machine. (**a**) shows the 0° FMA and (**b**) shows 29.1° FMA.

Figure 1, Figure 2, Figure 3, Figure 4, Figure 5 and Figure 6 show the design processes of the novel bio-engineered testbed, which are presented in three sections (1) bio-inspired design principles (Figure 1 and Figure 2); (2) Instron set-ups (Figure 3 and Figure 4); (3) Zwick–Roell set-ups (Figure 5 and Figure 6).

Section 1—Bio-inspired design principles.

Section 2—Instron set-ups.

Section 3—Zwick–Roell set-ups.

Section 1 includes 3D CAD models of the drug-releasing chewing robot based on a SOMSO skeleton model of a human skull as a biological pair [70] (SOMSOMODELLE GmbH, Adam Rouilly, Kent, UK) which passed a scientific accuracy test [48]. The cephalometric analysis (CeA) (Figure 1a) is also labelled with anatomical features including the Frankfurt horizontal plane (FHP), mandibular plane (MP) and Curve of Spee, which represented the bite force or power stroke on the second molar. The occluded pair of mandibular and maxillary molars with the integrated FMA in the removable chewing chamber are presented in Figure 1b. The Curve of Spee passes through the buccal cusp tips of the molars to the cusp tips of the mandibular molars. The Curve of Spee is regarded as the most important of Andrew’s six key principles, which is the plane of occlusion which should be horizontal or along the Curve of Spee [64], as shown in Figure 1a. The two bilateral functional springs in Figure 1b represent the temporalis muscles. They attach to each side of the maxillary jaw to hold the mechanical mandible in the correct occlusion and balance the torque generated during chewing whilst making contact with the mandible condyles and cam-driven mechanism. Figure 2 represent further kinematic information on the design process. Within Section 2, Figure 3a shows the various designs and the associated adaptors with 0° FMA, whilst Figure 3b shows CAD models with 29.1° FMA and a bite force or power stroke represented on the second molar. Figure 4 shows the manufactured and assembled testbed. Figure 5 and Figure 6 provide similar representations for the Zwick–Roell set-ups in Section 3.

### 2.2. Two-Bite Test–Texture Profile Analysis (TPA)

TPA is a popular double compression test for determining textural or mechanical properties in food science [49] and the pharmaceutical industry for oral drug release [28,36,37,41,50,51]. The TPA test is often called the “two-bite test” because the texture analyser intends to mimic the mouth’s biting action [71].

**Figure 7:**
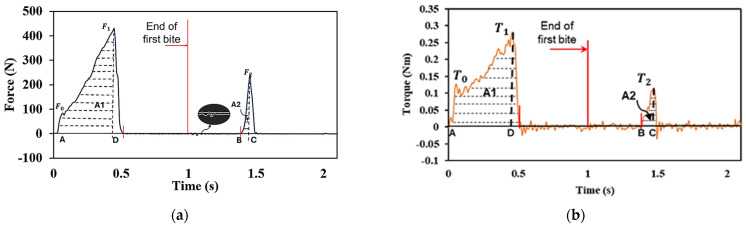
(**a**) Force–time graph from a two-bite test for MCGs using occluded molar pair with 25° FMA (+/− 5° is within normal human range); (**b**) torque–time graph from a two-bite test for MCGs using occluded molar pair with 25° FMA. Each bite takes one second as indicated by the red line on both graphs and the arrow shows the end of first bite.

The tests can be conducted with universal testing machines or texture analysers [49,50,51,52,72,73,74,75,76,77,78,79,80,81,82,83,84] where a top plate compresses a specimen under a particular compressive strain. The increasing force is measured via a load cell, then the top plate returns to its original value, followed by a second compression stage to the same position as the first stage. A force–displacement or force–time response graph is generated that correlates with the mechanical properties of food [49,52,72,73,74,75,76,77,78,79,80,82,85,86,87] or MCGs [37,41,42,50,83,84,88]. The TPA parameters can be derived either directly or indirectly from the response graph and be classed as primary or secondary. In Figure 7a, the initial highest peak in the first cycle is related to the hardness of the tested materials, $F_{1}$. The appearances of minor peaks during the first cycle that precede the hardness peak are indicators of the brittleness (i.e., a material’s tendency to fracture under stress with little or no plastic deformation before breaking) of the compacted material, $F_{0}$. The strength of the internal bonds in tested materials is defined as cohesiveness, which is calculated from the ratio of the positive areas under the graph for the first and second cycles, $\frac{A2}{A1}$. The mechanical behaviour of the tested material after the release of the applied force and the retraction of the compression probe can be related to their springiness/elasticity, which can be estimated from the time ratio between the initial points of the two cycles, $\frac{t_{B-C}}{t_{A-D}}$. Chewiness is a secondary parameter that could also be derived from the measured parameters; it is calculated as the multiplication of hardness, cohesiveness and springiness, $F_{1}\;$× $\frac{A2}{A1}$ × $\frac{t_{B-C}}{t_{A-D}}$. Compressibility is the ratio between the thicknesses of the tested samples before and after the two cycles of compression. It can be estimated as a percentage of the distances between A and D, $d_{A-D}$, and between B and C, $d_{B-C}$, using $\frac{{d_{A-D}+\;d}_{B-C}}{Original\;thickness}$. The parameters cohesiveness, springiness and chewiness relate to mastication physiology (i.e., the mechanical breakdown of food). Elevated values of these textural parameters could increase chewing time, jaw movement and muscle activity [85]. In particular, the springiness and cohesiveness provide important structural information [85]: cohesiveness (i.e., the force required to sever internal bonds), chewiness (i.e., the energy required to chew a product until is ready to be swallowed) [89] and compressibility indicates how easily food can be compressed during chewing. Adhesiveness, which is a textural property more appropriate to the stickiness of foods, is defined as the force value required for a probe to overcome the attractive forces (sticking) between its surface and the surface of the product being investigated [37,41,42,49,50,52,71,87,88,89,90,91]. It is represented by the negative area under the x-axis of the force–time graph at the end of the first bite—as enlarged in Figure 7a. Finally, the work done is determined by summing the positive areas under the force–displacement graph for each of the two highest force peaks, $A_{1}$ + $A_{2}$. This sum represents the energy required to deform the sample during the test [71].

In this study, the work done is more representative of human chewing in terms of bite force (or power stroke) and energy required to disrupt mechanical bonds. Bite force is primarily measured perpendicular to the Curve of Spee, and on the second molar is typically between 20 and 30° from the vertical (z) axis for hominids [61]. The bite force angle from the z-axis has been measured as 27.1°—as shown within Figure 1a. The resultant force acting on the maxilla when the jaws occlude is often at an angle to the direction of bite force and has two components: crush and shear. Pure crush forces are in-line with the bite force direction and are therefore orthogonal to the occlusal plane, which is the direction of pure shear forces. The bite force angle, θ, was measured as 72.2° which is similar to the human range that is between 55 and 80° [61].

Osborn [61] described the crush/shear ratio as the ratio of the orthogonal forces on the molar’s occlusal plane for human chewing. In our study, we propose for the first time, a method to accurately abstract the FMP plane and its geometrical relationship between the occluded pair of mandibular and maxillary molar teeth—as shown in Figure 1a—and the bio-engineered FMA in the testbed—as shown in Figure 3b and Figure 5. By measuring force and torque during the two-bite test, the crush/shear ratio can be calculated in terms of work done/energy required or power stroke as shown in Equation (1).

(1) $$\frac{Crush}{Shear}Ratio=\frac{Work\;done\;by\;crush\;force}{Work\;done\;by\;shear\;force+work\;done\;by\;torque}$$

Equation (1) is used alongside the TPA parameters to prove the critical role of jaw physiology and dental morphology on the experimental results when using in vitro apparatus, and moreover to evaluate the novel testbed and propose a framework for parameter optimisation.

Figure 7b shows a torque–time graph for MCGs, representing the molars shearing, crushing and grinding during a two-bite test. This sequence of three different masticatory processes is called the “power stroke of mastication”, which Simpson [92] defined into two phases. In phase I, the first movement (shearing) is on the buccal side (towards the cheek). This involves the shearing of food by the lower molars moving upwards and lateral to a centric occlusion point, which causes the cusps of the teeth to slide past each other to tear food apart. The second movement (crushing) is on the lingual side (towards the tongue). This involves the lower teeth moving directly upwards, causing the basins of the molars to enter the basins of the opposing molars, which crushes the food. In phase II, the final movement (grinding), involves the mandible moving laterally with a partial azimuth rotation, resulting in the cusps and basins of the molars sliding past each other and grinding food [93,94].

The chewing gum sample used in all experimental tests was Wrigley’s Extra White (The Wrigley Company, Plymouth, UK) which was used as a test sample for material uniformity with average dimensions of (22.4 × 10.8 × 6.5 mm). This was the MCG of choice for in vitro and in vivo clinical trials, containing xylitol as the investigative agent [10].

Figure 7 shows the response graphs from a universal testing machine illustrating the general principles of the two-bite test for the 25° FMA (+/− 5° is within normal human range) [95].

The experimental data is presented in Section 3.1, Section 3.2, Section 3.3, Section 3.4 and Section 3.5 with varying Instron and Zwick–Roell set-ups and with the objective of evaluating the bio-engineered testbed. There are five Set-ups (as shown below), where Set-up A utilises the Instron machine (without torque measurement) and the others utilise the Zwick–Roell (with torque measurement). The compression rate was set at 75% of the MCG thickness for both testing machines [73]. The experiments did not incorporate any artificial saliva or temperature.

Set-up A: Instron using 10 mm/s compression and FMA = 0 to evaluate tooth morphologies (including an occluded pair of molars).

Set-up B: Zwick–Roell using 10 mm/s compression and BA = 0 whilst varying FMA.

Set-up C: Zwick–Roell using 49.5 mm/s compression and BA = 0 whilst varying FMA (to emulate human chewing speed).

Set-up D: Zwick–Roell using 10 mm/s compression and BA = 8 whilst varying FMA (to evaluate the change in BA).

Set-up E: Zwick–Roell using 49.5 mm/s compression and BA = 8 whilst varying FMA (to emulate human chewing speed).

## 3. Results

### 3.1. Set-Up A: Instron Using 10 mm/s Compression and FMA = 0 to Evaluate Tooth Morphologies (Including an Occluded Pair of Molars)

**Figure 8:**
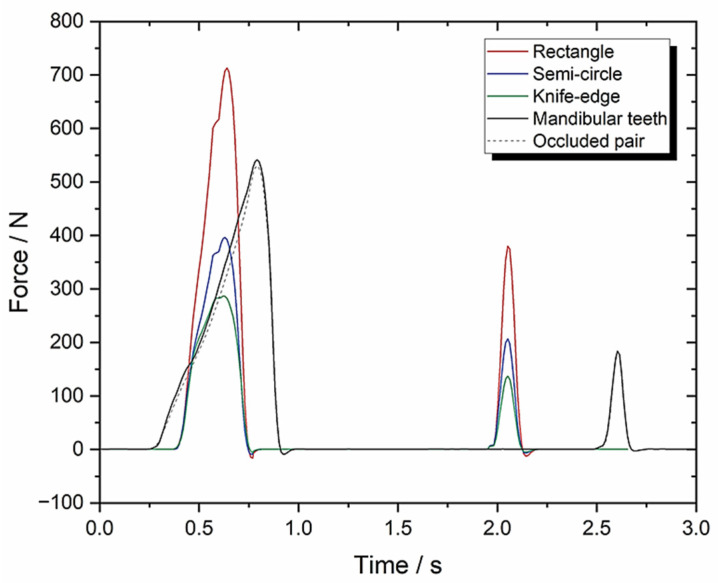
Set-up A: force-time graph with 10 mm/s compression, 0° FMA and 0° BA.

Figure 8 shows the force–time response graph and its associated TPA parameter calculations (Table 1). In this instance, the work done is calculated by $\;(A_{1}+$ $A_{2})$ rather than Equation (1) because the Instron machine is not capable of measuring torque.

### 3.2. Set-Up B: Zwick–Roell Using 10 mm/s Compression and BA = 0 Whilst Varying FMA

**Figure 9:**
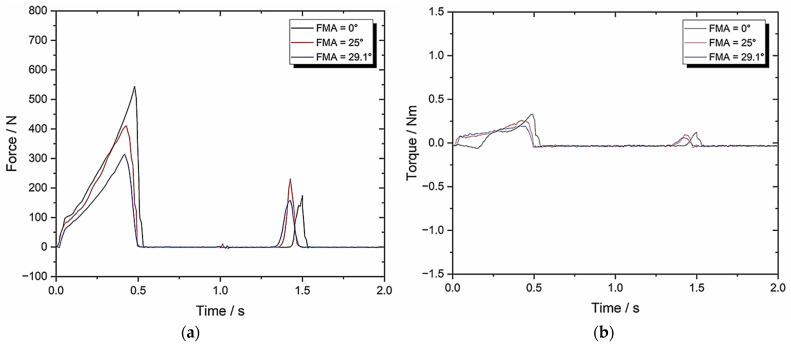
Set-up B: (**a**) force–time graph with 10 mm/s compression and 0° BA; (**b**) torque–time graph with 10 mm/s compression and 0° BA.

Figure 9 shows the resultant graphs and Table 2 contains the associated TPA calculations.

### 3.3. Set-Up C: Zwick–Roell Using 49.5 mm/s Compression and BA = 0 Whilst Varying FMA (To Emulate Human Chewing Speed)

**Figure 10:**
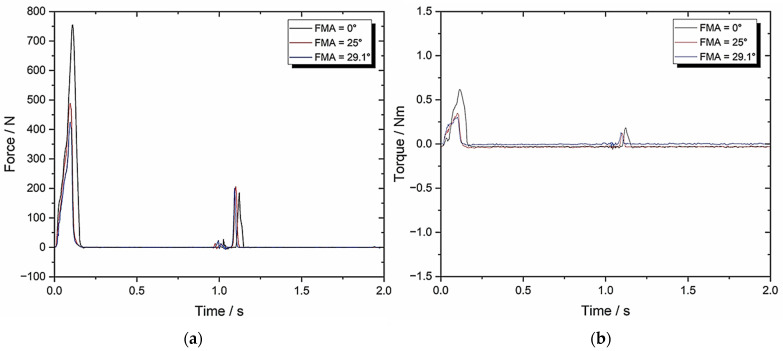
Set-up C: (**a**) force–time graph with 49.5 mm/s compression and 0° BA; (**b**) torque–time graph with 49.5 mm/s compression and 0°.

Figure 10 shows the response graphs and Table 3 contains the TPA parameters.

### 3.4. Set-Up D: Zwick–Roell Using 10 mm/s Compression and BA = 8 Whilst Varying FMA (To Evaluate the Change in BA)

**Figure 11:**
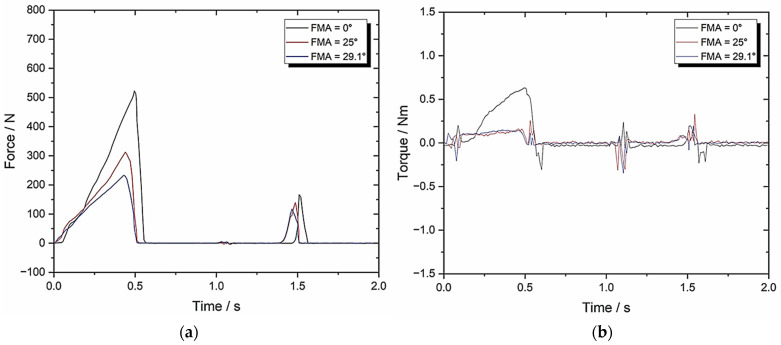
Set-up D: (**a**) force–time graph with 10 mm/s compression and 8° BA; (**b**) torque–time graph with 10 mm/s compression and 8° BA.

Figure 11 shows the response graphs and Table 4 contains its TPA parameters.

### 3.5. Set-Up E: Zwick–Roell Using 49.5 mm/s Compression and BA = 8 Whilst Varying FMA (To Emulate Human Chewing Speed)

**Figure 12:**
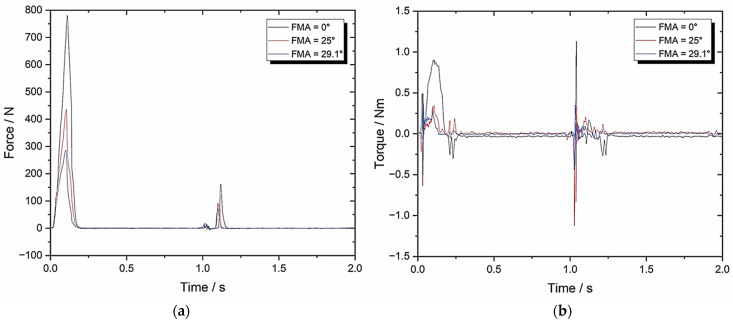
Set-up E: (**a**) force–time graph with 49.5 mm/s compression and 8° BA; (**b**) torque–time graph with 49.5 mm/s compression and 8° BA.

Figure 12 shows the resultant graphs and Table 5 contains the associated TPA calculations.

## 4. Discussion

Beginning with Set-up A, Figure 8 shows the force–time response graph and its associated TPA parameter calculations (Table 1). The objective was to demonstrate that human replica teeth, particularly the occluded pair of molars were more effective for chewing in terms of morphology than other, simpler geometries, as shown in Figure 3.

As a reference, Al Hagbani et al., 2018 [50] tested the mechanical properties of three commercial chewing gums, Wrigley^®^, Extra^®^ and Nicotine^®^. A TA-XT plus texture analyser was fitted with a flat-faced TA-4 cylindrical probe and used a constant compression of 4.0 mm/s. They found average values of cohesiveness (%) (0.48 ± 0.08); springiness (%) (0.28 ± 0.04); chewiness (N) (6.18 ± 0.79) and compressibility (%) (0.92 ± 0.08). Cohesiveness and chewiness are defined as primary and secondary parameters which are a measure of the force required to disrupt mechanical bonds and the energy required to chew a product until is ready to be swallowed, respectively. These two TPA parameters are used alongside springiness/elasticity (i.e., mechanical behaviour parameters) for direct comparison.

With a flat-faced morphology, Set-up A produced eight times the value of chewiness (48.27 ± 2.41), similar springiness/elasticity (0.33 ± 0.02) and half the compressibility (0.52 ± 0.03), respectively, when compared with the reference.

With a mandibular teeth morphology, Set-up A produced twice the value of chewiness (11.9 ± 0.6), lower springiness/elasticity (0.242 ± 0.012) and lower compressibility (0.75 ± 0.038), respectively, when compared with the reference. Table 1 provides clear evidence for the relationship between work done and teeth morphology when transitioning from primitive shapes to complex morphologies.

The data generated for the occluded pair of molars is closely comparable to the reference values except for the cohesiveness result (0.06 ± 0.003) which is much higher in Al Hagbani et al., 2018 [50]. This parameter is associated with the strength of internal bonds and it demonstrates the effectiveness of the power stroke from occluded pair of molars to break mechanical bonds. The lower springiness (0.225 ± 0.011) means that the tooth morphology requires less mastication energy. Moreover, cohesiveness, springiness and chewiness relate to mastication physiology (i.e., the mechanical breakdown of food); these three parameters have the lowest values for the occluded pair of molars when compared with other tooth shapes in Table 1.

For Set-up B using the Zwick–Roell device, Figure 9 shows the resultant graphs and Table 2 contains the associated TPA calculations. The two objectives were to compare the set-ups using FMA = 0, and then to evaluate how increasing the FMA affects biting forces with respect to the crush/shear ratio and the work done.

When using an occluded pair of molars with FMA = 0, both set-ups generate similar results, although the springiness and chewiness are slightly lower (0.225 ± 0.011)/(0.1 ± 0.005) and (7.31 ± 0.37)/(2.79 ± 0.14), respectively. However, the sample size for Set-up B was lower (3 rather than 10) which does provide a less representative average.

After increasing the FMA from 0 to 25, there is a noticeable difference in some of the TPA parameters. Specifically, the hardness decreases by 33%, the chewiness increases by 256%, and the work done is reduced by 35%. Raising the FMA from 25 to 29.1 generates another noticeable decrease in the hardness and work done values, which results in a lower crush/shear ratio (4.22 to 3.41).

This is clear evidence of the effectiveness of altering the FMA in terms of chewing efficiency and shows the importance of FMA during mastication—this is critical for drug release [10,45] and provides some validation for this bio-engineered testbed.

Figure 10 shows the response graphs for Set-up C and Table 3 contains the TPA parameters. The objective was to identify any changes to the results after increasing the compression speed to 49.5 mm/s which is comparable to human chewing speed. The main difference was in the chewiness value, which is far higher—the remaining parameters were elevated slightly, but there was no change in the crush/shear ratio when increasing the FMA.

Figure 11 shows the response graphs and Table 4 contains the TPA parameters for Set-up D. The objective was to identify the effect of BA on the calculated mechanical properties.

In comparison to Set-up B, which implemented BA = 0, the springiness and chewiness with BA = 8 are very similar when FMA = 0, (0.11 ± 0.005)/(0.1 ± 0.005) and (3.2 ± 0.16)/(2.79 ± 0.14), even with the sample size increased to eight. However, once the FMA rises to 25 and 29.1, clear reductions in the hardness are visible (by 21% and 37%, respectively), which in turn reduced the overall work done (by 19% and 20%, respectively).

This is clear evidence that demonstrates the importance of BA on chewing efficiency.

Furthermore, Set-up E (Figure 12, Table 5) demonstrated the importance of BA at chewing speeds comparable to humans. Figure 12b clearly illustrates that greater torque values (when compared with Figure 10b) are imposed on the MCG during the two-bite test which, combined with lower chewiness values, generated lower work done and thus an improved chewing efficiency. This is particularly true at higher FMAs, which yields more evidence of the impact of both the FMA and the BA on TPA parameters and further validates the novel two-bite testbed in terms of chewing efficiency and power stroke.

In all the experimental set-ups, the adhesiveness value had been very low. This parameter is calculated from the negative area under the x-axis at the end of the first bite (as indicated in Figure 2). Kaushik et al., 2024 [96] optimised batches of MCG-2 containing promethazine for drug release using TPA and in vitro study with apparatus A. They identified that chewing gum with low adhesiveness values (0.10) was given a positive evaluation since the gum particles give the cud a nice chew because they do not stick to the denture or to themselves [96]. Unfortunately, Al Hagbani et al., 2018 [50] did not use the adhesiveness parameter in their analysis.

These extensive experimental results are clear evidence illustrating the impact that jaw physiology, dental morphology and form–function have on TPA parameters. Consequently, there are benefits of in vitro masticatory apparatus, as highlighted in Section 1. For MCG drug release, apparatus A and B had limited jaw anatomical physiology, as highlighted by Stomberg et al., 2018 [44] and Externbrink et al., 2019 [46]. As mentioned earlier in Section 1, Stomberg et al., 2018 attempted to improve apparatus B by developing a 45º gliding steel unit with a four-cylindrical-cusped molar to imitate the occlusal plane of natural dentition [44]. Despite the authors’ valiant attempts, the improvement to apparatus B had limited anatomical accuracy, and the occlusal surface geometries and mastication was very simplified. This was already addressed by Alemzadeh et al., 2020 [10], who created a novel chewing robot with built-in humanoid jaws.

For dental science, Krueger et al., 2021 [14] tested the applicability of Artificial Resynthesis Technology (ART 5) to create microwear textures under controlled conditions, replicating the oral environment. In their two experimental chewing studies where they used occluding pairs of third molars that were surgically extracted, one study used dried beef and another study added sand to the dried beef. Their preliminary results showed that ART 5 as a dental simulator produces microwear textures and concluded that these results indicate its potential to untangle the complex variables of dental microwear formation. A more thorough experimental protocol would be necessary with ART 5 to improve controls, the pH of the simulated oral environment and grit measurements. It is interesting to note that occluding pairs of third molars were used in their study. In this study, more pairs of molars (M1, M2 and M3) were integrated which is a more realistic representation of the human jaw and is beneficial for food fracturing in vitro apparatus [6] or even two-bite tests.

For food science, as highlighted in Section 1, the comprehensive review of systematic designs over the past 15 years by Guo et al., 2024 [19] highlighted the current limitations of the physiological structures of masticatory simulators, stating that there are no established methods/criteria for evaluating their biomimetic components. The manuscript clearly addresses testing methods, standardising chewing efficiency and the criteria for evaluating biomimetic components [19] with the PLM framework with the built-in reverse biomimetics methodology developed by Alemzadeh [48].

Walker et al., 2022 [20] investigated the effect of dental morphology, form–function and comminution chewing experiments in catarrhines using the BA chewing simulator developed by Salles et al., 2007 [6] for food fracturing. The size of the molars used in the chewing simulator were all equal due to the simplification of some aspects of mastication (i.e., as a trade-off), knowing that tooth size has an influence on the fragmentation of certain types of food, in terms of mechanics. They proposed that other simulators under development [10,14,24,25] may allow further studies on the form–function relationships of teeth. This relationship is investigated by integrating the occluded pair of molars into the bio-engineered testbed based on the chewing robot with built-in humanoid jaws developed by Alemzadeh et al., 2020 [10].

Within Section 3, the results clearly illustrate the effectiveness of jaw physiology (i.e., FMA and BA) and tooth morphology with respect to chewing efficiency and effective mastication for drug release from MCGs, when transitioning from primitive shapes to complex morphologies even without artificial saliva and temperature in all experiments (Set-ups A–E). The data generated from the results can be optimised with the presence of artificial saliva and temperature in a further study. The major advantage and benefit of the novel bio-testbed is that it can be used with universal testing machines and provides a better framework and future direction for two-bite tests.

To this date, new apparatus is still constantly being developed based on biomimetics and bio-inspired designs [97].

## 5. Conclusions

A novel bio-engineered two-bite testbed was created for two universal testing machines, with compression and torsion, based on bionics design principles for accurate comminution and molars intercuspation and effective mastication, in terms of mechanochemistry and power stroke to break mechanical bonds.

Extensive experimental studies with different setups for the FMA and the BA [63] were conducted to systematically analyse a two-bite test to evaluate the mechanical properties of MCGs, and to probe the form–function relationships between biting force and crushing/shearing aspects in order to understand chewing efficiency and the effective power stroke of mastication. The manuscript critically analysed the impact and importance of jaw physiology, dental morphology and the FMA and the BA on two-bite test parameters, validating the bio-engineered testbed for API release from MCGs. This work also has impact in other scientific fields such as dental science, food science and the pharmaceutical industries for drug release that use in vitro masticatory apparatus, as critically analysed and discussed in Section 1 and Section 4. This proof-of-concept (POC) chewing platform could be used to validate drugs such as aspirin, caffeine, CPP-ACP, dimenhydrinate and chlorhexidine contained within commercially available MCG and administered for pain relief, as a central-nervous-system (CNS) stimulant, tooth decay, motion sickness and dental hygiene, respectively.

Our innovative approach combined the fields of bionics, bioengineering, dental biomechanics and biomedical engineering to address a significant barrier in the commercial development of MCGs [10,48]. The research group’s vision is to use and optimise this enabling technology to test tailored drug release from MCGs to determine API doses that would be received by patients in a controlled, safe and user-friendly manner, that would also reduce development costs. This advancement in drug delivery technology has the potential to improve patient care and outcomes as functional chewing gums for health are growing in popularity and the market is expected to reach USD 3.18 billion by 2028 [98].

## 6. Patents

Dental simulator—Kazem Alemzadeh https://patents.google.com/patent/US20090035739A1/en (accessed on 31 May 2025).

## Figures and Tables

**Table 1:** Set-up A parameters with 10 mm/s compression, 0° FMA and 0° BA (*n* = 10).

TPA Parameters	Knife-Edge	Rectangle or Flat	Semi-Circle	Mandibular Teeth	Occluded Pair of Molars Teeth
Hardness (N)	287.20 ± 14.36	713.51 ± 35.68	396.32 ± 19.82	546.5 ± 27.33	541.7 ± 27.09
Cohesiveness	0.16 ± 0.008	0.205 ± 0.010	0.18 ± 0.009	0.09 ± 0.005	0.06 ± 0.003
Springiness	0.29 ± 0.015	0.33 ± 0.02	0.28 ± 0.014	0.242 ± 0.012	0.225 ± 0.011
Chewiness	13.33 ± 0.67	48.27 ± 2.41	19.97 ± 1	11.9 ± 0.6	7.31 ± 0.37
Compressibility	0.44 ± 0.022	0.52 ± 0.03	0.44 ± 0.022	0.75 ± 0.038	0.827 ± 0.041
Work Done (J)	40.74 ± 2.04	91.07 ± 4.55	54.17 ± 2.71	1480.5 ± 74.03	1448.67 ± 72.43

**Table 2:** Set-up B parameters with 10 mm/s compression and 0° BA (*n* = 3).

TPA Parameters	0° FMA	25° FMA	29.1° FMA
Hardness (N)	558.01 ± 27.90	419.88 ± 21	318.87 ± 15.94
Cohesiveness	0.05 ± 0.003	0.10 ± 0.005	0.12 ± 0.006
Springiness	0.10 ± 0.005	0.17 ± 0.009	0.20 ± 0.01
Chewiness	2.79 ± 0.14	7.14 ± 0.36	7.65 ± 0.38
Compressibility	0.82 ± 0.041	0.87 ± 0.044	0.89 ± 0.045
Max Torque (Nm)	0.31 ± 0.016	0.24 ± 0.012	0.17 ± 0.009
Work Done (J)	1295.05 ± 64.75	958.67 ± 47.93	709.18 ± 35.46
Crush to Shear Ratio	∞	4.22 ± 0.21	3.41 ± 0.17

**Table 3:** Set-up C parameters with 49.5 mm/s compression and 0° BA (*n* = 8).

TPA Parameters	0° FMA	25° FMA	29.1° FMA
Hardness (N)	760.12 ± 38.01	496.54 ± 24.83	430.85 ± 21.54
Cohesiveness	0.09 ± 0.005	0.17 ± 0.009	0.19 ± 0.01
Springiness	0.29 ± 0.015	0.21 ± 0.011	0.22 ± 0.011
Chewiness	19.84 ± 1.0	17.73 ± 0.887	18.01 ± 0.9
Compressibility	0.92 ± 0.046	0.83 ± 0.042	0.86 ± 0.043
Max Torque (Nm)	0.59 ± 0.03	0.33 ± 0.017	0.3 ± 0.015
Work Done (J)	1301.71 ± 65.09	1524.69 ± 76.23	1421.68 ± 71.08
Crush to Shear Ratio	∞	4.22 ± 0.21	3.41 ± 0.17

**Table 4:** Set-up D parameters with 10 mm/s and 8° BA (*n* = 8).

TPA Parameters	0° FMA	25° FMA	29.1° FMA
Hardness (N)	581.66 ± 29.08	347.69 ± 17.38	232.59 ± 11.63
Cohesiveness	0.05 ± 0.003	0.09 ± 0.005	0.10 ± 0.005
Springiness	0.11 ± 0.005	0.27 ± 0.014	0.23 ± 0.012
Chewiness	3.2 ± 0.16	8.45 ± 0.42	5.35 ± 0.27
Compressibility	0.83 ± 0.042	0.95 ± 0.048	0.91 ± 0.046
Max Torque (Nm)	0.65 ± 0.033	0.19 ± 0.01	0.19 ± 0.01
Work Done (J)	1292.67 ± 64.63	805.15 ± 40.26	590.48 ± 29.52
Crush to Shear Ratio	26157 ± 1307.85	4.22 ± 0.21	3.41 ± 0.17

**Table 5:** Set-up E parameters with 49.5 mm/s compression and 8° BA (*n* = 8).

TPA Parameters	0° FMA	25° FMA	29.1° FMA
Hardness (N)	793.47 ± 39.67	446.75 ± 22.34	294.36 ± 14.72
Cohesiveness	0.06 ± 0.003	0.11 ± 0.006	0.13 ± 0.007
Springiness	0.19 ± 0.01	0.23 ± 0.012	0.23 ± 0.012
Chewiness	9.04 ± 0.45	11.3 ± 0.57	8.8 ± 0.44
Compressibility	0.84 ± 0.042	0.84 ± 0.042	0.85 ± 0.043
Max Torque (Nm)	0.96 ± 0.048	0.45 ± 0.023	0.38 ± 0.019
Work Done (J)	1971.80 ± 98.59	1123.21 ± 56.16	791.09 ± 39.55
Crush to Shear Ratio	19634 ± 981.7	4.22 ± 0.21	3.41 ± 0.17

## Data Availability

The data presented in this study are available on request from the corresponding author due to privacy.

## Abbreviations

The following abbreviations are used in this manuscript:

MCG	Medicated chewing gum
PLM	Product lifecycle management
API	Active pharmaceutical ingredient
BA	Bennett angle of mandible
FMA	Frankfort-mandibular plane angle
TMJ	Temporomandibular Joint
CUR	Curcumin
TPA	Texture profile analysis
CeA	Cephalometric analysis
FHP	Frankfurt Horizontal plane
MP	Mandibular Plane
ART	Artificial Resynthesis Technology
CNS	Central Nervous System
POC	Proof of Concept
CPP-ACP	Casein Phosphopeptide-Amorphous Calcium Phosphate
